# Calcium Fluoride

**Published:** 1909-05-15

**Authors:** 


### Therapeutics and Pharmacy.

### CALCIUM FLUORIDE.

The therapeutic uses of the fluorides have received comparatively little attention, notwithstanding the fact that it is now generally recognised that a certain amount of fluorine in the form of calcium fluoride is a necessity for the proper strengthening and consistence of the bones and for the normal hardness and good condition of the teeth. According to the *Pharmaceutical Journal*, however, at least two observers have successfully employed calcium fluoride therapeutically—namely, A. Robin and A. Brissemoret. The powder originally prescribed by Robin is said to have been as follows:—

| | |
|---|---|
| Magnesium carbonate ... ... | 10 cgm. |
| Calcium carbonate ... ... ... | 25 cgm. |
| Calcium triphosphate ... ... ... | 25 cgm. |
| Calcium fluoride ... ... ... | 1 cgm. |
| White sugar ... ... ... ... | 1 gram. |

One such powder to be taken daily.

Brissemoret found that the administration of calcium fluoride to the extent of 5 milligrams per diem, 15 days a month, had a marked influence in arresting dental caries. He advocates its use for growing children if the bones do not seem very strong; for children or adults suffering from dental caries; and for cases of fracture to promote the formation of callus; also for tuberculosis to aid the remineralisation of the system, and during pregnancy and lactation under certain conditions. In these cases he prescribes the following powder:—

| | |
|---|---|
| Calcium fluoride ... ... ... | 75 milligrams. |
| Potassium phosphate ... ... | 3 grams. |
| Sodium phosphate ... ... ... | 5 grams. |
| Magnesium phosphate ... ... | 10 grams. |
| Calcium phosphate ... ... ... | 10 grams. |
| Sodium citrate ... ... ... | 15 grams. |
| Milk sugar ... ... ... | to 100 grams. |

Half a teaspoonful of the mixed powder twice daily, at meals.

For cases of fractures, to hasten the formation of callus, and to strengthen it when formed, the following powder is given:—

| | |
|---|---|
| Calcium fluoride ... ... ... | 5 cgm. |
| Magnesium fluoride ... ... ... | 2 cgm. |
| Calcium bromide ... ... ... | 2.5 grams. |
| Calcium phosphate ... ... ... | 5 grams. |
| Calcium carbonate ... ... ... | 5 grams. |

Mix and divide into 20 powders. One twice daily.

We do not pretend to vouch for the efficacy of the above prescriptions, still less of the fluorides contained in them; but we think that the suggestions seem sound and worthy of consideration. We should be glad to hear the experiences of any readers who have tried this method of treatment.

